# SARS-CoV-2 in lions, gorillas and zookeepers in the Rotterdam Zoo, the Netherlands, a One Health investigation, November 2021 

**DOI:** 10.2807/1560-7917.ES.2023.28.28.2200741

**Published:** 2023-07-13

**Authors:** Florien Dusseldorp, Linda G.R. Bruins-van-Sonsbeek, Maaike Buskermolen, Henk Niphuis, Mariëlle Dirven, Jane Whelan, Bas B. Oude Munnink, Marion Koopmans, Ewout B. Fanoy, Reina S. Sikkema, Aimée Tjon-A-Tsien

**Affiliations:** 1 Public Health Services Rotterdam Rijnmond, the Netherlands; 2 Rotterdam Zoo, Rotterdam, the Netherlands; 3 Biomedical Primate Research Centre, Rijswijk, the Netherlands; 4 Department of Viroscience, Erasmus MC, WHO Collaborating Centre for Arbovirus and Viral Hemorrhagic Fever Reference and Research, Rotterdam, the Netherlands

**Keywords:** SARS-CoV-2, zoo, public health, occupational health, zoonosis, outbreak investigation, One Health

## Abstract

In November 2021, seven western lowland gorillas and four Asiatic lions were diagnosed with COVID-19 at Rotterdam Zoo. An outbreak investigation was undertaken to determine the source and extent of the outbreak and to identify possible transmission routes. Interviews were conducted with staff to identify human and animal contacts and cases, compliance with personal protective equipment (PPE) and potential transmission routes. Human and animal contacts and other animal species suspected to be susceptible to SARS-CoV-2 were tested for SARS-CoV-2 RNA. Positive samples were subjected to sequencing. All the gorillas and lions that could be tested (3/7 and 2/4, respectively) were RT-PCR positive between 12 November and 10 December 2021. No other animal species were SARS-CoV-2 RNA positive. Forty direct and indirect human contacts were identified. Two direct contacts tested RT-PCR positive 10 days after the first COVID-19 symptoms in animals. The zookeepers’ viral genome sequences clustered with those of gorillas and lions. Personal protective equipment compliance was suboptimal at instances. Findings confirm transmission of SARS-CoV-2 among animals and between humans and animals but source and directionality could not be established. Zookeepers were the most likely source and should have periodic PPE training. Sick animals should promptly be tested and isolated/quarantined.

**Key public health message**

**What did you want to address in this study?**

In Rotterdam Zoo, in late 2021, two animal species (gorillas and lions) were simultaneously infected with SARS-CoV-2, despite the use of personal protective equipment (PPE) by their zookeepers. We aimed to identify all possible transmission routes and provide recommendations to optimise the protection of animals and zookeepers from infection.

**What have we learnt from this study?**

We found several possible transmission paths and SARS-CoV-2 positive zookeepers were the most likely source of the outbreak. The genomic data of SARS-CoV-2 supported transmission of SARS-CoV-2 among animals and between humans and animals. It was beneficial to work with several specialists in animal and human health, virology, occupational medicine, public health and zoo management during the outbreak investigation.

**What are the implications of your findings for public health?**

This outbreak affecting multiple species showed challenges in preventing interspecies SARS-CoV-2 transmission. To prevent respiratory zoonotic diseases, it is necessary to adopt stringent prevention and control strategies in zoos. Working with animals may make adhering to PPE guidelines challenging. Zookeepers should be supported in using PPE correctly, by providing repeated training and by provision of PPE that fits optimally.

## Background

To date (March 2023), the COVID-19 pandemic, caused by severe acute respiratory syndrome coronavirus 2 (SARS-CoV-2), resulted in more than 600 million cases and 6.5 million human deaths worldwide [1]. The common hypothesis is that SARS-CoV-2 originated from bats and was transmitted to humans through an intermediate animal host [2-4]. Severe acute respiratory syndrome coronavirus 2 has a broad host range, as shown by the numerous animal species (to date 26 species, all mammals) that have been infected with SARS-CoV-2 [5,6]. In some cases, SARS-CoV-2 circulation in animal populations such as hamsters, minks and white-tailed deer, has led to infection of humans in contact with infected animals [7-10].

In several zoos, SARS-CoV-2 has caused infections among captive animals such as felines and non-human primates, likely caused by human-to-animal transmission [11-13]. To protect zoo animals and their zookeepers, a One Health approach is crucial to identify possible transmission routes of SARS-CoV-2 between animals and humans.

### Outbreak detection

In November 2021, within 6 days, multiple western lowland gorillas and Asiatic lions experienced fever, coughing and lethargy in Rotterdam Zoo, the Netherlands. An outbreak was confirmed when both animal species were diagnosed with COVID-19 by means of positive SARS-CoV-2 real-time polymerase quantitative chain reaction (RT-qPCR) tests. The risk of further transmission among animals, staff and visitors were reasons to conduct an extensive outbreak investigation.

In this report we describe the findings of investigating the source, possible transmission routes and the extent of the outbreak.

## Methods

### Setting and COVID-19 infection prevention measures

The Rotterdam Zoo welcomed 1.5 million visitors per year before the pandemic [14]. The zoo covers an area of 28 hectares and houses over 600 animal species. Currently there are 185 employees, of which 55 are involved in the direct care of animals. During the period of the outbreak (November 2021), the zoo was open for visitors.

The lions and gorillas each have indoor facilities and adjacent outdoor enclosures, their enclosures are ca 200 m apart (Supplementary Figure S1). The outside enclosure of the gorillas is surrounded by a few metres of bushes. At some spots where the public can come closer, the gorillas are separated from the public by means of a ditch and a glass wall. Gorillas hardly use their outdoor enclosure during autumn and winter. The indoor gorilla enclosures have a separate airflow and the enclosure is separated by a wall and glass window from the visitors. There is an open connection between the indoor gorilla enclosures and the staff work- and breakrooms (via an open roof construction of the gorilla enclosure and an air vent and possible open windows of the breakroom of the staff). In the lion indoor enclosure, air from the visitors’ area flows through a high-efficiency particulate air (HEPA) filter through the indoor enclosures of the animals to outside. The lions’ and gorillas’ food was prepared in different kitchens.

When SARS-CoV-2 emerged in 2020, zookeepers were instructed to use additional preventive measures when caring for primates or felines according to the Dutch Zoo Federation (NVD) protocol [15]. Personal protective equipment (PPE) was used when caring for the animals where sufficient distancing was not possible. Personal protective equipment included a filtering facepiece particle 2 (FFP2) mask, face shield, gloves and boots. Boots were sanitised with a disinfectant (Virkon-S) when entering and leaving the animal enclosures. Zookeepers were tested for SARS-CoV-2 in case of COVID-19-like symptoms. They were offered COVID-19 vaccination under the Dutch national vaccination programme, which started in January 2021.

Visitors were obliged to show a COVID-19 admission ticket i.e. a proof in the form of a QR code that they had been (i) fully vaccinated either with two doses of Comirnaty, Spikevax or Vaxzevria, Moderna Vaccine or one dose of Jcovden, (ii) recovered from COVID-19, or (iii) recently tested negative for SARS-CoV-2. Moreover, a one-way walking route for visitors was set up in the zoo. It was possible for visitors to throw food, although not allowed, to the outdoor animal enclosures.

### Epidemiological investigation

An outbreak team was convened with the zoo head veterinarian, public health and communication experts of the Public Health Service (PHS) and virologists specialised in One Health.

### Contact tracing and case definition

Direct contact was defined as contact of > 15 min at < 1.5 m with persons or animals who tested SARS-CoV-2 positive. Indirect contact was defined as having entered the gorilla or lion enclosures or being involved with animal food preparation or having come into contact with animal faeces, all regardless of the use of PPE. Clinical and epidemiological data were collected on the identified human and animal cases. A case was defined as RT-qPCR-confirmed SARS-CoV-2 infection in either animals or staff between 2 weeks before and 4 weeks after the first occurrence of symptoms compatible with a COVID-19 infection of an animal. A line-listing of all identified cases was compiled and summarised according to time, place and person.

### Microbiological investigation

Specimen collection and diagnostics

Nose and throat specimens were collected from identified contacts, including all zookeepers (n = 19) of the gorillas and lions and facility staff members (n = 21) at 3 and 5 days after the confirmation of SARS-CoV-2 in those animals respectively. The samples were then analysed for SARS-CoV-2 in regional laboratories.

Samples were taken from lions and gorillas, where possible, after the first clinical signs of disease. After confirmation of SARS-CoV-2 infection, all animals of the taxonomic (sub)orders of Primates and Carnivora were sampled between 19 November and 8 December 2021. Following novel reports on SARS-CoV-2 infections in white-tailed deer and hippopotamuses, faeces of related animal species were also tested for SARS-CoV-2 if possible (Supplementary Table S1) [16,17]. Samples were also taken from black and rufous elephant shrews because one was found dead and the necropsy revealed hyperaemic lungs. Animal specimens included individual nose and throat swabs, nasal discharge and faecal samples collected from animal enclosures.

Rodents that were caught in regular pest control between 15 November and 8 December 2021 were also tested. Specimens were pooled per animal species and throat swabs, rectal swabs and lung tissue of all rodents were tested for SARS-CoV-2 RNA [18]. Lung tissue was retrieved from the mice and rats and stored dry until processing. A piece of tissue of ca 4 × 4 mm was cut off and transferred into a vial with a ceramic bead and 300 µL MagNA Pure 96 DNA Tissue Lysis Buffer (Roche Diagnostics GmbH, Mannheim, Germany). Total nucleic acid isolation from the swab material was performed on MagNA Pure 96 (Roche Molecular Systems Inc, Branchburg, United States (US)). All swab specimens were tested in a duplex PCR reaction on SARS-CoV-2 and PDV, where SARS-CoV-2 primers were targeting the E-gene and RdRp gene.

#### Serology

To study the extent of the SARS-CoV-2 outbreak, available sera (blood drawn for other purposes) collected between October 2021 and March 2022 were tested for IgG antibodies for SARS-CoV-2. We did not subject the animals to blood sampling for outbreak research. The procedure for the direct enzyme-linked immunosorbent assays (ELISA) is as previously described [19] but with recent modifications. The virus antigens used in the ELISA to screen sera for antibodies reactive or cross-reactive to SARS-COV-2 was a 1:1 mixture of AG-COVID-19-S1/-S2 (XpressBio, Frederick, US). The coating solution consisted of 1.0 mg virus antigen/ml Tris-NaC1-EDTA Buffer, 0.25 % triton X-100. Fifty µl were added to each well of ELISA plates (half area, 96 well, Greiner Bio One, Frickenhausen, Germany), at a concentration of 2  µg/ml in phosphate-buffered saline and incubated overnight at room temperature. AG-COVID-19-S1 and AG-COVID-19-S2 (XpressBio) mixtures (1:1) were used as antigens in ELISAs to screen for antibodies reactive or cross-reactive to human SARS-COV-2 virus. The rest of the procedure was performed according to Ogunro et al. [19]

#### Whole genome sequencing

Severe acute respiratory syndrome coronavirus 2-positive specimens with sufficient viral loads were sequenced using an amplicon-based Nanopore sequencing approach, using ARTIC V3 primers [20]. Libraries were generated using the native barcode kits from Nanopore (EXP-NBD196 and SQK-LSK109) and sequenced on a R9.4 flow cell multiplexing up to 96 samples per sequence run. Sequence data analysis was performed as previously described [21].

All available genomes from the Netherlands in the period February 2020 to September 2021 were downloaded from GISAID and down sampled based on 10 nucleotides (nt) differences [22]. All available full-length (> 90% coverage) SARS-CoV-2 genomes were included in the analysis (retrieved from GISAID on 30 November 2021) and aligned with the SARS-CoV-2 sequences from this study. The alignment was manually checked for discrepancies, after which IQ-TREE was used to perform a maximum-likelihood phylogenetic analysis under the GTR + F + I + G4 model as the best predicted model using the ultrafast bootstrap option with 1,000 pseudoreplicates [23].

### Environmental assessment

To investigate other routes of transmission, an extensive environmental investigation was initiated. The zoo veterinarian was interviewed concerning the functioning of the sewage system, the food handling, possibilities of (in)direct contact between visitors and animals, pests and the occurrence of incidents that may have enabled transmission between humans and animals. Public Health Service infectious disease specialists visited the zoo to assess compliance to hygiene measures, PPE use in practice [15] and the animal enclosures (including their ventilation systems) to identify risks for SARS-CoV-2 transmission via droplets or airborne.

## Results

### Clinical infections in gorillas and lions

**Figure 1:**
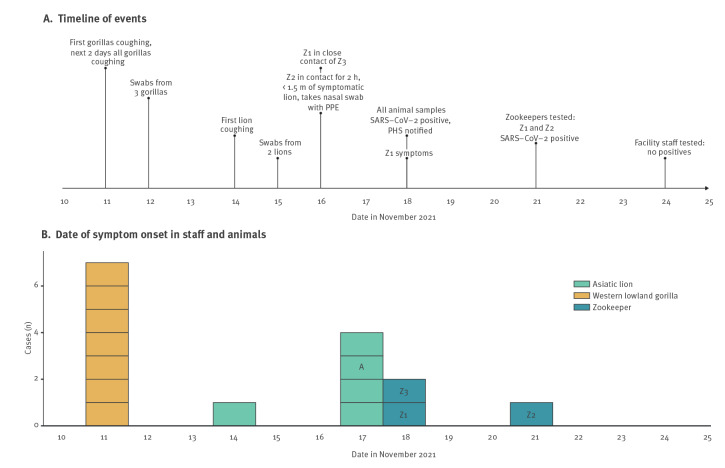
Chronological events summarising the COVID-19 outbreak in gorillas and lions, Rotterdam Zoo, the Netherlands, November 2021

On 11 November 2021, zookeepers noticed that some of the gorillas were coughing. In the next 2 days, all seven gorillas living in the same enclosure displayed coughing and a decreased food intake and three of the seven gorillas also showed sweating and lethargy (Figure 1). An eighth gorilla living in a separate enclosure did not display any symptoms. On 14 November, one lion displayed lethargy, and 3 days later four of five lions displayed coughing, anorexia and lethargy. One young female did not have symptoms. Specimens of faeces and nasal secretions from three of seven gorillas and two of four lions were collected between 12 and 15 November 2021. On 18 November 2021, these were confirmed SARS-CoV-2 positive by RT-qPCR (Supplementary Table S2). In addition, faecal samples deriving from multiple gorillas or lions (group samples) also tested positive. The animals tested negative for influenza, parainfluenza and rhinovirus and *Toxocara*.

A: asymptomatic; Z1: zookeeper for gorillas; Z2: zookeeper for lions; Z3: zookeeper 3 (this zookeeper tested positive on 18 November and it is unknown whether this person experienced symptoms; Z 3 had no direct or indirect contact with gorillas or lions during the outbreak).

COVID-19 onset date for the gorillas was uncertain. On Day 0 the zookeepers noticed coughing but could not establish which gorilla was coughing. In the next 2 days all gorillas started to display symptoms.

We assumed that the asymptomatic lion cub (A) was SARS-CoV-2 positive and thus indicated the same symptom onset date as her mother and siblings.

For the asymptomatic zookeeper of the lions (Z2), the date of the positive SARS-CoV-2 test is indicated.

The data are graphically illustrated in an epidemiological curve incorporated in a timeline, in which the events are described chronologically (Figure 1). One week after disease onset in the gorilla group, the gorillas started to improve clinically. They recovered within 2 weeks. Also 1 week after disease onset, the lions improved clinically. They were symptom-free after 19 days. None of the zoo animals were vaccinated against SARS-CoV-2.

### Epidemiological investigation

#### Contact tracing

The gorillas and lions were managed under two different animal departments and were taken care of by separate groups of zookeepers. Both groups used the same changing rooms. Nineteen staff were identified as direct contacts: two veterinarians, a trainee veterinarian, eight gorilla zookeepers, six lion zookeepers and two zoo laboratory staff. Twenty-one facility staff were identified as indirect contacts e.g. through food handling and cleaning of the animal housing.

Seven gorillas in one enclosure and a solitary gorilla in a different enclosure were identified as direct contacts of each other and/or their zookeepers. Four lions lived with each other, and another adult female was housed separately from the group, however, they shared the same enclosure using a rotating system. Consequently, the lion group and the single female were indirect contacts via secretions of each other. Three mangabeys living in the outside gorilla enclosure were considered direct contacts of the gorillas.

### Microbiological investigation

#### RT-qPCR

To identify the possible source of infection in the animals, direct human contacts were RT-qPCR tested on 21 November 2021. Two of 19 were confirmed SARS-CoV-2 positive: one zookeeper of the gorillas (Z1) and one of the lions (Z2). Since the zookeepers had high viral loads (quantification cycle (Cq) Z1: 19.5, Cq Z2: 22.5) and one had symptom onset after the start of illness among the animals, the source of the outbreak remained uncertain. Therefore, we decided to test all but one (one staff member declined) indirect contacts (n = 20) on 24 November 2021, all of whom tested negative. The solitary living gorilla and mangabeys also tested negative. All faecal samples of all other animals that are part of the orders Carnivora (n = 17), Primates (n = 70), Macroscelides (n = 1) and Ungulates (n = 5) tested negative (Supplementary Table S1).

#### Serology

Seventy-one serum samples of 13 different species belonging to the orders Carnivora, Primates and Ungulates (Carnivora n = 5, Primates n = 5, Ruminants n = 5, Pachyderms n = 56) were screened for IgG antibodies for SARS-CoV-2 between October 2021 and March 2022 (Supplementary Table S3). None of the sera was positive. There was no serum available for animals who tested RT-qPCR positive after November 2021. The serum sample collected on 9 March 2022 belonged to the gorilla who lived in the separate enclosure.

#### Whole genome sequencing

**Figure 2:**
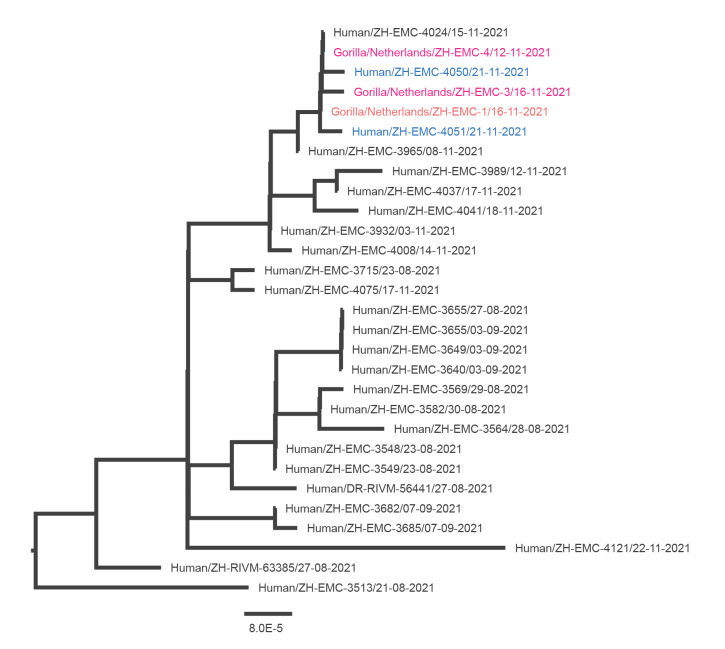
Zoom-in of the phylogenetic analysis, including two zookeepers (Z1 and Z2) and two gorillas of Rotterdam Zoo, and other Delta variants of SARS-CoV-2 found in the same period and province in the Netherlands, August–November 2021

Z1: zookeeper for gorillas; Z2: zookeeper for lions.

All samples from positive humans and animals were subjected to whole genome sequencing (WGS) (Figure 2). Whole genome sequencing was successful for two zookeepers and two gorillas (100% genome coverage). For the two lions, 50% genome coverage was accomplished due to low viral loads (Cq value lion 1 faeces: 29.7, Cq values lion 2 saliva: 37.2, nasal discharge 35.7). Sequence analysis indicated that these four SARS-CoV-2 strains belonged to the Delta variant (Phylogenetic Assignment of Named Global Outbreak (Pango) lineage designation (B.1.617.2). The gorillas were both infected with an identical virus. When resampled 4 days later, the full sequence of one infected gorilla showed one mutation compared with the first sample (hCoV-19/gorilla/Netherlands/ZH-EMC-4/2021 vs hCoV-19/gorilla/Netherlands/ZH-EMC-3/2021 Figure 2). One partial sequence of a sample of an infected lion had 1 nucleotide difference from the sequences of infected gorillas and zookeepers, while the other was identical.

Human sequences are blue, gorilla sequences are red. Of one gorilla, two sequences from different time points were obtained. The consensus sequences are deposited in GISAID [22] under the accession numbers EPI_ISL_7650924, EPI_ISL_7650929, EPI_ISL_7651169, EPI_ISL_8377349 and EPI_ISL_8377350 and the raw sequence data are available in the European Nucleotide Archive (ENA) under the accessions SAMEA12219786, SAMEA12219787, SAMEA12219788, SAMEA12219789, SAMEA11232778 and SAMEA11232779.

### Characteristics of human cases

Ultimately, three zookeepers were confirmed SARS-CoV-2 positive. Zookeeper Z1 who was taking care of gorillas, had symptom onset on 18 November 2021. This zookeeper had contact with zookeeper Z3 on 16 November, who tested positive on 18 November. Zookeeper Z3 had not been in contact with the gorillas, the lions or with zookeeper Z2 (Supplementary Table S4). Zookeeper Z2 taking care of lions, remained asymptomatic, and tested positive on 21 November.

### Environmental assessment

No sewage defects were reported during the outbreak. There was no water surrounding the animal enclosures that could mix with wastewater. Small rodents such as mice could enter the animal enclosures. Fifteen rodents were caught in regular pest control between 15 November and 8 December 2022. In total, eight mice and seven rats were caught in the zoo, of which five rodents were caught in or around the gorilla enclosure and one in the lion enclosure. None of the caught rodents tested positive for SARS-CoV-2 RNA. It was impossible to reach the animal enclosures for larger wild and feral animals.

**Figure 3:**
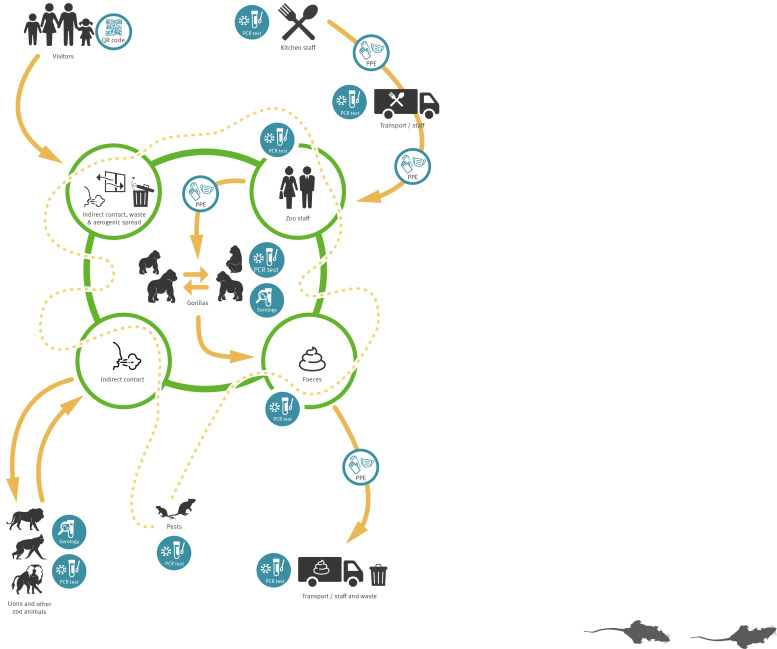
Graphical display of possible SARS-CoV-2 transmission routes

Due to heat and discomfort, zookeepers reported touching their facemask sometimes while cleaning and facemasks were incidentally re-used. We listed the probability of hypothetical transmission routes based on known literature and expert opinion and the results of the outbreak investigation in the Table and displayed it graphically in Figure 3.

**Table:** Hypotheses sources of SARS-CoV-2 infection in Asiatic lions and western lowland gorillas, Rotterdam Zoo, the Netherlands, November 2021

Hypothesis	Route	Source	Probability	Comments
Human to animal	Direct (droplets/airborne)	Zookeepers	Most likely	An earlier asymptomatic infectious case among the zookeepers may have been missed. In the internal enclosures, zookeepers wearing facemasks could have come in close contact with lions or gorillas. Staffrooms were adjacent to the gorilla enclosure and connected via an open connection.
Human to animal	Direct (droplets/airborne)	Visitors	Unlikely	The distance between visitors and animals outside is at least 1.0 m. Indoor enclosures are fully closed (glass/wall), separating animals from visitors. For the lions, air of visitors flows via an HEPA filter through the indoor enclosure of the lions to the outside.
Human to animal	Indirect (fomites)	Zookeepers/facility staff	Less likely	20 facility staff members tested SARS-CoV-2 negative on 24 November 2021 and an asymptomatic infectious case among the facility staff may have been missed. One staff member did not take the test. Transmission may have occurred during cleaning of the enclosures or during food preparation.
Human to animal	Indirect (fomites)	Visitors	Unlikely	Visitors may have thrown, or the wind may have blown, contaminated rubbish or food leftovers into the animal exhibits. Evolving evidence about SARS-CoV-2 transmission suggests that the share of transmission of SARS-CoV-2 via fomites is small [25].
Sewage	Waterborne (faeces) or fomites	Sewage	Highly unlikely	SARS-CoV-2 virus was detected in regional sewage, however, no problems with sewage (e.g. flooding of surface water nearby outside enclosures) were detected.
Intermediate host	Fomites, faeces or droplets	Other wild animals such as rodents, mustelids, bats, feral cats, squirrels, foxes etc. (1–3).	Highly unlikely	15 mice and rats were caught in the zoo, of which five rodents were caught in the gorilla and lion exhibits. All tested negative for SARS-CoV-2 RNA. It was impossible to reach the animal enclosures for most other (larger) wild and feral animals.

#### Infection prevention and control measures

After the first confirmed SARS-CoV-2 cases among animals, immediate control measures to limit the outbreak were initiated: the gorillas and lions were isolated and shielded off from the visitors until the animals fully recovered (after 19 days). The hygiene protocol was extended to the use of changing overalls and splash goggles.

## Discussion

We describe the results of an extensive investigation of a SARS-CoV-2 outbreak in a zoo among lions and gorillas. It is most probable that the animals were infected through their zookeepers, although we could not determine the transmission routes with certainty. In this outbreak, two animal species, which were housed ca 200 m apart from each other and cared for by two different zookeeper groups, plus two direct human contacts (zookeepers), were infected, despite PPE use and the implemented preventive measures of the zoo. 

Listing all hypothetical transmission routes, we considered one or multiple asymptomatic infectious zookeepers, who may have had contact with each other in private settings or in the changing rooms, as the most likely outbreak source. Subsequent animal-to-animal transmission is likely given the high attack rate among the animals and the consistent PPE use of the zookeepers. Moreover, the genomic data of the two zookeepers and the lions and gorillas clustered, which may indicate transmission between the animals and their zookeepers. However, similar Delta variant SARS-CoV-2 circulated predominantly in the Rotterdam population during the same time. Therefore, it is impossible to make definitive statements on the outbreak source, based on the generated sequence data. Previous SARS-CoV-2 infections in zoo-managed animals also pointed to zookeepers as the most probable source of infection [11-13,24]. These reports did not mention the compliance of the zookeepers regarding PPE use.

Other possible outbreak routes are airborne or indirect transmission (e.g. fomites or food-borne) to the animals by infectious visitors. However, this is unlikely since the distance between visitors and animals outside is at least 1 m and evolving evidence about SARS-CoV-2 transmission suggests that the share of SARS-CoV-2 transmission via fomites is small [25]. In a recent outbreak report, 12 animals from six species contracted SARS-CoV-2 in a zoo in Chicago (US) [26]. In this study, by MC Allender et al, genomic data suggested human-to-animal transmission and although zookeepers were not proven to be the source, they could not be ruled out either [26]. SARS-CoV-2 material was found exclusively on the inside of the air filter, indicating that despite viral particles possibly making it to the air filtration system, they were not being circulated. Indoor SARS-CoV-2 transmission from visitors to animals through air conditioning is therefore also unlikely.

In a zoo, the number of captive animals living in proximity to each other is relatively small, which makes the chance of developing a new variant of SARS-CoV-2 in an animal reservoir small. However, knowledge on transmission and identifying potential reservoirs is important for monitoring and surveillance purposes.

Before this outbreak, no animals had been confirmed to be SARS-CoV-2 infected in our zoo. The absence of positive serologic findings supports the absence of previous SARS-CoV-2 infections. A systematic review showed that after vaccination, neutralising antibodies are detectable after 1 week in primates [27]. This may explain why we did not see a positive serology response on a sample of an infected gorilla drawn on 16 November (5 days after the beginning of the outbreak).

The two identified SARS-CoV-2 infected staff displayed symptoms and tested positive 7 days after the disease onset of their animals. They were also found to have high viral loads, indicating a recent infection. Therefore, we considered them an unlikely source of the SARS-CoV-2 infection of the animals. Although human-to-human transmission is the most probable explanation, we cannot rule out animal-to-human transmission. The zookeepers may have (self) contaminated through touching their facemasks because of discomfort (trouble breathing, heat) and their reported re-use of facemasks. Besides that, both zookeepers had close contact with the sick animals.

Working with animals may make adhering to PPE guidelines challenging. Challenges with PPE, such as uncomfortable facemasks during physically tough work in warm circumstances or inadequate doffing and donning, which is also seen with healthcare professionals, may play a role in several outbreaks that have been reported among captive animals and their human contacts worldwide [11-13,28-30]. After the implementation of the additional infection and prevention measures, no new SARS-CoV-2 cases in animals of the Rotterdam Zoo were detected despite increased surveillance. To limit risks for respiratory zoonoses in the future, an PHS infection prevention specialist attended the zoo to give additional PPE training to the zookeepers.

Based on our experience and the literature on the topic, we propose five actions to limit the risk of SARS-CoV-2 transmissions in zoos. Firstly, all animals in zoos showing clinical COVID-19-like signs should be promptly tested for respiratory pathogens [30]. Secondly, species known to be susceptible to SARS-CoV-2 and where animal-to-human transmission has previously been described should be included in contact investigations and be quarantined swiftly. Thirdly, we recommend supporting zookeepers in using PPE correctly by providing both periodic training and PPE with optimal fitting. Management should recognise that correct handling of PPE may affect work efficiency of staff. Fourthly, during a period of high transmission in the regional population, the potential transmission paths between visitors and zoo animals should be critically evaluated. Possible measures taken may include: (i) increasing distance between visitors and animals; (ii) placing glass walls instead of fences where increasing the distance is not possible; and (iii) checking indoor ventilation systems. Lastly, animals known to be SARS-CoV-2 susceptible may be considered for vaccination [31]. However, at this time, European zoo veterinarians do not see the urge to vaccinate since according to our experience animals generally show mild signs of the disease (Wildlife Health (ZWH) conference of the European Association of Zoo and Wildlife Veterinarians (EAZWV), 24–28 May 2022, panel discussion ‘SARS-CoV-2 vaccination, 27 May 2022, Wildlands, Emmen the Netherlands) [32]. The proposed recommendations may also apply to other settings and other respiratory pathogens, in which humans are in an occupational setting in close contact with large groups of animals such as farms and petting zoos.

## Conclusion

We described an outbreak of COVID-19 among two animal species in separate locations within a single zoo, in which positive zookeepers were the most probable source of the outbreak despite PPE use by them. We identified several potential transmission paths. It is crucial to adopt stringent prevention and control strategies to avoid introduction of respiratory pathogens in animal populations. One Health collaboration remains important to contribute to the reduction of known and potential public health risks and occupational hazards for zoo staff.

## Supplementary Data

289000 Supplementary Material
