# Supplementary material for: SARS-CoV-2 in lions, gorillas and zookeepers in the Rotterdam Zoo, the Netherlands, a One Health investigation, November 2021
Source: Euro Surveill. 2023 Jul 13;28(28):2200741. doi: 10.2807/1560-7917.ES.2023.28.28.2200741 (PMC10347891; doi:10.2807/1560-7917.ES.2023.28.28.2200741)

Supplementary

This supplementary material is hosted by *Eurosurveillance* as supporting information alongside the article 'SARS-CoV-2 in lions, gorillas and zookeepers in the Rotterdam Zoo, the Netherlands, a One Health investigation, November 2021' on behalf of the authors, who remain responsible for the accuracy and appropriateness of the content. The same standards for ethics, copyright, attributions and permissions as for the article apply. Supplements are not edited by *Eurosurveillance* and the journal is not responsible for the maintenance of any links or email addresses provided therein.

Supplementary Table S1: Samples submitted to SARS-CoV-2 RT-PCR

**Animal species**	**Sample type**	**Date**	**No. specimens**	**No. animals in Zoo**
Amur leopard (*Panthera pardus orientalis*)	Feces (individual)	19-11-2021	1	2
Francois' langur (*Trachypithecus francoisi*)	Feces (group)	19-11-2021	1	4
Gelada (*Theropithecus gelada)*	Feces (group)	19-11-2021	2	11
Western lowland gorilla (*Gorilla gorilla gorilla)*	Nose swabs, throat swabs, feces (individual), feces (group)	(12-30) - 11-2021 ; 10-12-2021	32	8
Crested black macaque (*Macaca nigra*)	Feces (group), nose swab, throat swab	19-11-2021; 4-1-2022	1	5
Lions *(Panthera leo persica)*	Nasal discharge, nose swab, feces (individual), saliva	(15;20) - 11-2021	4	5
Mangabey *(Cercocebus lunulatus*)	Feces (group)	19-11-2021	2	8
Palas' cat (*Otocolobus manual)*	Feces (group)	19-11-2021	1	2
Ring tailed lemur (*Lemur catta)*	Feces (group)	(19;23) -11-2021,	15	19
Serval (*Leptailurus serval)*	Feces (individual, group)	19-11-2021	2	4
Tiger (*Panthera tigris sumatrae)*	Feces (individual)	19-11-2021	1	2
Lion-tailed macaque (*Macaca silenus)*	Nose swab, throat swab, feces (group)	23-11-2021	3	4
Black-and-rufous elephant shrew *(Rhynchocyon petersi)*	Nose swab, throat swab,	23-11-2021	2	5
Cotton-top tamarin *(Saguinus oedipus)*	Feces (group) nose swab, throat swab, rectal swab	24-11-2021; 8-12-2021	5	7

Okapi, (*Okapia johnstoni)*	Feces (individual)	14-1-2022	5	5
Giraffe (*Giraffa camelopardalis reticulata)*	Feces (group), feces (individual)	(19 – 22) -1-2022	3	7
Southern pudu (*Pudu puda)*	Feces (individual)	19-1-2022	2	2
Bongo (*Tragelaphus eurycerus*)	Feces (individual)	22-1-2022	1	2
Red panda (*Ailurus fulgens)*	Feces (group), feces (individual)	2-12-2021	3	3

Supplementary table S2: Ct-values of sampled animals

**Animal**	**Sample date**	**Sample type**	**CT (E-gen)**
Gorilla 1	12-11-2021	Nose swab	23.0
Gorilla 2	12-11-2021	Nose swab	28.1
Lion 1	15-11-2021	Faeces	29.7
Lion 2	15-11-2021	Saliva	37.2
Gorilla group sample	15-11-2021	Faeces	39.1
Gorilla 2	16-11-2021	Nose swab	19.7
Gorilla 2	16-11-2021	Throat swab	20,0
Gorilla 1	16-11-2021	Nose swab	20.4
Gorilla 2	18-11-2021	Faeces	33.7
Gorilla group sample	19-11-2021	Faeces	32.0
Lion group	19-11-2021	Saliva	35.1
Lion 2	20-11-2021	Nasal discharge	35.7
Gorilla group	22-11-2021	Faeces	33.8

Supplementary Table S3: Samples submitted to SARS-CoV-2 ELISA

**Animal species**	**Date**	**No. specimens**	**No. of animals in Zoo**
Ring tailed lemur (*Lemur catta)*	19-02-2021; 15-03-2021	2	15
Malayan tapir (*Tapirus indicus)*	01-10-2021; 02-10-2021; 28-12-2021	3	2
Eastern black rhinoceros (*Diceros bicornis michaeli)*	(04;11;18;19;25)-10-2021; (1;8;10;12;15;22;29)-11-2021; (6;13;20;23;28)-12-2021	43	4
Southern pudu (*Pudu puda)*	11-10-2021	1	2
Artic Fox (*Vulpes lagopus)*	12-10-2021	1	2
Western lowland gorilla (*Gorilla gorilla gorilla)*	15-10-2021; 16-11-2021; 09-03-2022	3	8

Asian elephant (*Elephant maximus)*	27-10-2021; (25;29)-11-2021; (07;09)-12-2021	10	6
White naped mangabey (*Cercocebus lunulatus)*	01-11-2021	1	9
Raccoon (*Procyon lotor)*	03-11-2021; 30-11-2021	2	6
Blackbuck (*Antilope cervicapra)*	10-11-2021; 13-12-2021	2	13
Mhorr gazelle (*Nanger dama mhorr)*	15-11-2021	1	5
Okapi (*Okapi johnstoni)*	01-12-2021	1	5
Cotton top tamarin (*Saguinus oedipus)*	02-12-2021	1	7

Supplementary Table S4: Characteristics of the 2 SARS-CoV-2 positive human contacts of the animals

**Characteristic**	**Zookeeper 1**	**Zookeeper 2**
Date of positive test	November 21, 2021	November 21, 2021
Ct value	19.5	22.5
Date of onset of symptoms	November 18, 2021	Not applicable
Symptoms	Coughing, sore throat, nasal cold and headache	Asymptomatic
Vaccination status	Vaccinated with Pfizer BioNTech (2x)	Vaccinated with Pfizer BioNTech (2x)
Time since vaccination	Approximately 2 months	Approximately 3 months
Animals	Mammal department 1, which includes gorillas.	Mammal department 2, which includes lions.
Interaction with SARS-CoV-2 infected animals	Daily contact within <1.5 meter with gorilla's for administering medication and food.<br>Daily cleaning of exhibits with regular garden hose and wearing FFP2 + full face mask.<br>A sick gorilla had coughed into the zookeepers face at November 11, the zookeeper was wearing a medical face mask, glasses and gloves at that moment.	Three times a day contact with lions, administered medication.<br>Zookeeper swabbed a lion at November 16, and cleaned lion exhibit for 2 hours, the lion was (behind a fence) within 1.5 meter.
Reported particularities in use of PPE	Sometimes re-use of masks.<br>Takes facemask off sometimes due to heat/discomfort during cleaning.	Sometimes re-use of masks.
Known contact with a SARS-CoV-2 positive person?	Yes, at 18-11 with Zookeeper 3 (this zookeeper did not have direct or indirect contact with lions or gorilla's during the outbreak and therefore was not tested during the test round on November 21)	No

Supplementary Figure S1: Map of Rotterdam Zoo with the lions exhibit, the gorilla exhibit and an overview of the tested species. The lion and gorilla exhibits are separated by a distance of approximately 200 m.

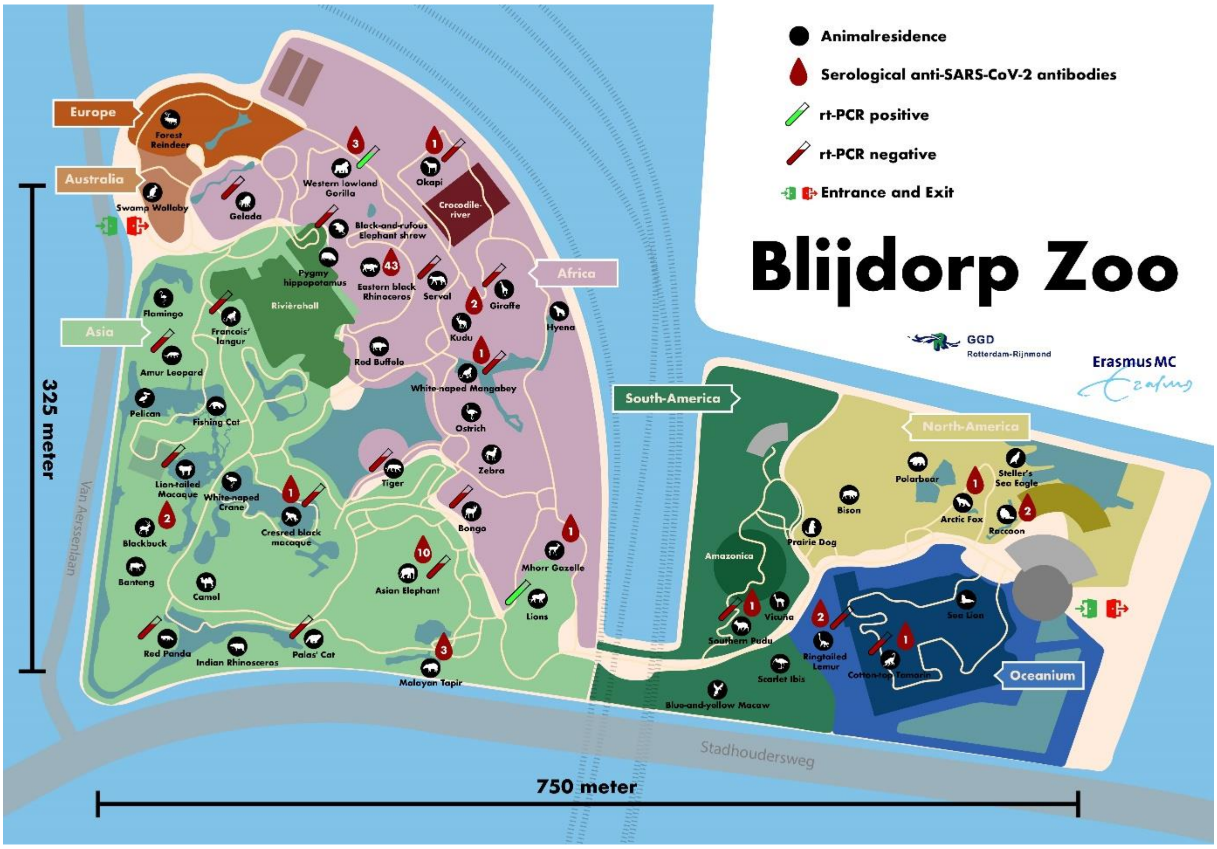

Supplement: Supplementary Material [file 22-00741_DUSSELDORP_Supplementary_material.pdf]
